# A comparison of oncologist versus mental health provider attitudes towards standardized and tailored patient-reported outcomes

**DOI:** 10.1186/s41687-021-00352-8

**Published:** 2021-08-24

**Authors:** Salene M. W. Jones, Aliana Gaffney, Joseph M. Unger

**Affiliations:** Fred Hutchinson Cancer Research Center, 1100 Fairview Ave N, Seattle, WA 98109 USA

**Keywords:** Questionnaires, Measurement-based care, Symptom monitoring

## Abstract

**Background:**

Patient-reported outcomes (PROs) can be used to monitor patients during treatment. Healthcare provider preferences for individualized vs. standardized PROs have been understudied.

**Methods:**

This study surveyed oncology and mental health providers to compare attitudes towards individualized and standardized PROs. We have developed a method for individualizing PROs, called precision PROs, and the survey specifically assessed preferences for this method. We compared attitudes and preferences by provider type and by whether respondents were current or never users of PROs.

**Results:**

Oncology providers expressed more positive attitudes for standardized PROs in treatment planning compared to mental health providers (F(1,440) = 5.978, p = 0.015). The interaction between provider type (oncology vs. mental health) and type of PRO (individualized vs. standardized) was not significant for the attitudes about the clinical utility of PROs (p = 0.709). When directly asked about the precision PRO approach, oncologists were less likely to prefer standardized items (OR = 0.478, p = 0.001) or have no preference (OR = 0.445, p = 0.007) to the precision PRO approach when compared to mental health providers. Qualitative analyses suggested standardized PROs may be simpler or easier to understand whereas individualized PROs better capture patient variability and the unique aspects of each patient’s condition. Some mental health providers expressed reticence about letting patients choose how to tailor PROs. Never users of PROs reported more positive attitudes towards individualized measures than standardized measures whereas current users of PROs did not have a difference in attitudes (p = 0.010). User status was mostly unrelated to preferences.

**Conclusion:**

Results suggest that healthcare provider preference for individualized PROs may differ by medical specialty. How PROs are tailored may need to differ by discipline. This is particularly important given that previous research showing a preference for individualized PROs over standardized was conducted with psychotherapists. Further research on patient preferences for individualized and standardized PROs is warranted as is research on the clinical utility of individualized PROs such as the precision PRO approach.

**Supplementary Information:**

The online version contains supplementary material available at 10.1186/s41687-021-00352-8.

## Introduction

Patient-reported outcomes (PROs) are questionnaire-based measures of symptoms and quality of life that come directly from the patient [1]. PROs are an integral part of measurement-based care (MBC) in several aspects of healthcare, including oncology and mental health [2–4]. MBC involves periodically measuring patients’ signs and symptoms, often with PROs, to determine if treatment is working or needs to be changed [5].

In mental health, there has been a long history of tension between nomothetic or standardized measures in which all patients complete the same items with the same interpretation guidelines and idiographic measures that are tailored and individualized to each patient but are difficult to compare between patients or to norms [6]. One reason for exploring methods of individualizing or tailoring PROs in the idiographic approach is the suggestion that these measures are more sensitive to change [7]. Initial research from counselors in 2017 suggested that healthcare providers might prefer PROs that are individualized or tailored to each patient’s values and condition [8].

We have developed a method that tailors PRO items and the meaningful change definition to support the use of individualized PROs, dubbed precision PROs [9]. The first part of the Precision PRO approach asks patients or participants to define a personal minimally important difference (MID) for defining treatment response. This personal MID has been tested in a general medical sample [10]. The second part of the Precision PRO approach asks patients or participants to choose which items or symptoms from a PRO are most meaningful to them personally so the content of the PRO is tailored to the individual patient. In addition to the potential issues of provider preference, we developed this method to address the problem with current MID and responder definitions not incorporating individual patient values and preferences [11]. We have recently completed a test of both Precision PRO approaches in people with cancer and pain. The Precision PRO approach defines the MID and treatment response by what is most valuable or meaningful to the individual patient.

The preference of healthcare providers for or against such individualized approaches has not been studied extensively and differences by medical specialty have not been explored. Most studies examine preference or attitudes for standardized, nomothetic methods [12]. To address this gap, we surveyed oncology and mental health providers to determine if preferences for individualized PROs differ by specialty. We also conducted exploratory analyses comparing current users of PROs to providers who never used PROs to determine if individualizing PROs might improve uptake among never users.

## Methods

### Participants and procedures

Participants were recruited through survey panels maintained by Qualtrics in August 2019. Participants’ status as either an oncology provider (OP) or mental health provider (MHP) was verified through their National Provider Identification number. Potential participants were sent an invitation to complete the survey online. When participants came to the survey link, they first read the consent form and then, if they agreed to complete the study, clicked through to the survey. Participants received standard incentives for completing the survey such as store gift cards and airline miles. Typical response rates for Qualtrics panels are 5–12%. All procedures were reviewed and approved by the Fred Hutchinson Cancer Research Center review board (#8703).

### Measures

#### Attitudes towards standardized and individualized assessment

To assess provider attitudes towards standardized and individualized PROs, participants completed two subscales from the Attitudes towards Standardized Assessment and Attitudes towards Individualized Assessment scales [8], two validated and reliable measures of provider attitudes. Each of these attitudes scales has a clinical utility subscale (8 items, 6 reverse scored) and a treatment planning subscale (5 items). Each item is rated on a five-point scale from strongly disagree to strongly agree. Subscale scores are created by averaging the items and we specifically only scored the measures if participants had responded to at least half the items for the subscale. The two scales have the same 13 items and two subscales except one references standardized measures and the other references individualized measures. For example, an item from the treatment planning subscale is “Standardized progress measures help identify when treatment is not going well” on the standardized version and “Individualized progress measures help identify when treatment is not going well” on the individualized version. Items are averaged with higher scores indicating more positive views of either standardized or individualized measures.

#### Preference for precision PRO approach

To assess participants preferences for the precision PRO approach specifically, two close-ended and two open-ended questions were asked (see Additional file 1). The standard PRO approach was based on traditional nomothetic approaches [6, 13]. The first close-ended question described the standard minimally important difference (MID) approach and precision PRO MID then asked participants which approach they preferred. The standard version was scored on a scale of 0 to 100, where an increase or decrease of five points was considered meaningful for all patients. The Precision PRO version was also scored on a scale of 0 to 100 but patients define for themselves what increase or decrease in symptoms is meaningful for the patient personally, sometimes in consultation with a physician. The second close-ended question described a standard symptom PRO and the precision PRO approach for tailoring items with participants again asked to indicate which they preferred. The standard version had 5 fixed items (symptoms) that were answered by all patients. In the precision PRO version, however, participants chose 5 items (symptoms) out of a list of 30 possible items or symptoms that were most applicable to them personally. For both close-ended questions, ‘don’t know’, ‘neither’ and ‘both’ options were provided (see Additional File 1). The two open ended questions asked participants for their reasons for the close-ended question responses.

#### Characteristics

The first question on the survey asked participants to indicate if they had never used PROs in treatment monitoring, currently use PROs, or used to use PROs but no longer do. Because few participants (n = 21) reported formerly using PROs, these participants were excluded from analyses on user type. However, former users were included in analyses examining provider type (oncology vs. mental health). Participants also reported various demographic and professional characteristics.

### Quantitative analysis

For the Attitudes toward Standardized/Individualized Assessment subscales, we conducted a series of analyses of covariance (ANCOVA). Provider type was a between-subjects factor and type of PRO (standardized, individualized) was a within-subjects factor in the first set of ANCOVAs. In the second set of ANCOVAs, user type (never vs. current) replaced provider type as the between-subjects factor. Age and gender were included as covariates. For the preferences questions, responses were coded into three categories: precision PRO preference (reference); standard PRO preference; and no preference (prefer both, don’t know, neither, skipped question). The three preference categories were used as the outcome variable in a multinomial regression. The predictor of interest for the first set of multinomial regressions was provider type while covarying for age and gender. The predictor of interest for the second set of multinomial regressions was user type (never vs. current) while covarying for age and gender.

### Qualitative analysis

After the preference questions, participants provided an explanation supporting their choice. The text for the explanations were coded by two members of the research team using a content analysis approach. The codebook was developed by coder 1 after reviewing the responses given by OPs and MHPs. Next, coder 2 coded each of the 450 responses for the individualized-items question into one or more of the 17 categories and coded each response for the individualized-MID question into one or more of the 15 categories. Coder 1 double coded 10% of responses for each question to ensure reliability. The two coders discussed major differences and agreed upon revisions to both the codebook and responses. Codes did not have to be mutually exclusive.

## Results

The demographics of the sample (n = 450) were consistent with being drawn from a healthcare provider population (Table 1). The average age was 51.1 years old and most participants were White (n = 313, 69.6%) or Asian (n = 107, 23.8%). Slightly more than two-thirds of the sample was male (n = 308, 68.4%). Most respondents currently used PROs to monitor treatment (n = 347, 77.1%).

**Table 1:** Sample description

Characteristic	Mean (SD) or N (%)
Age, years	51.1 (11.4)
Gender	
Male	308 (68.4%)
Female	129 (28.7%)
Other, declined to answer	13 (2.9%)
Race/Ethnicity	
White	313 (69.6%)
Black or African-American	8 (1.8%)
Hispanic	16 (3.6%)
Asian	107 (23.8%)
Native American, Pacific Islander, Other	15 (3.3%)
Provider type	
Mental Health	250 (55.6%)
Oncology	200 (44.4%)
Has a doctorate-level medical degree	418 (92.9%)
Has non-medical doctorate degree	47 (10.4%)
PRO use	
Never user	82 (18.2%)
Former user	21 (4.7%)
Current user	347 (77.1%)
Number of patients seen per week	81.3 (76.6)

### Quantitative analyses

#### Attitudes Towards Assessment Scale

**Fig. 1:**
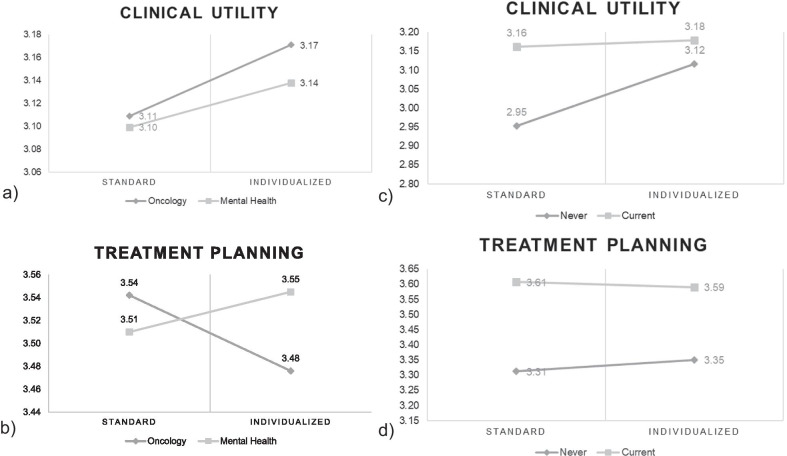
Means for the Attitudes towards Standardized and Individualized Assessment scales by provider type (**a**, **b**) and user type (**c**, **d**)

Overall, healthcare providers reported neutral attitudes towards assessment scales. Means ranged from 2.95 to 3.61 (Fig. 1) with 3 on the scale representing neither positive nor negative attitude towards assessment scales. Oncology and mental health providers did not differ in their attitudes towards assessment scales for clinical utility (F(1,442) = 0.139, p = 0.709; Fig. 1a). However, there was a significant interaction between provider type and standardized vs. individualized assessment in treatment planning (F(1,440) = 5.978, p = 0.015; Fig. 1b). Oncology providers tended to have more positive attitudes towards standardized assessments whereas mental health providers did not have a more positive attitude towards standardized assessments. For user type, there was a significant interaction between user type and standardized vs. individualized assessment in clinical utility (F(1,421) = 6.720, p = 0.010; Fig. 1c) such that never users tended to report more positive attitudes towards individualized measures whereas current users reported no difference in attitudes. For user type and treatment planning, there was a main effect in which current users reported more positive attitudes than never users (F(1,419) = 11.833, p = 0.001; Fig. 1d). No other main effects or interactions were significant (p’s > 0.05).

#### Direct comparison of precision PRO and standard approaches

**Fig. 2:**
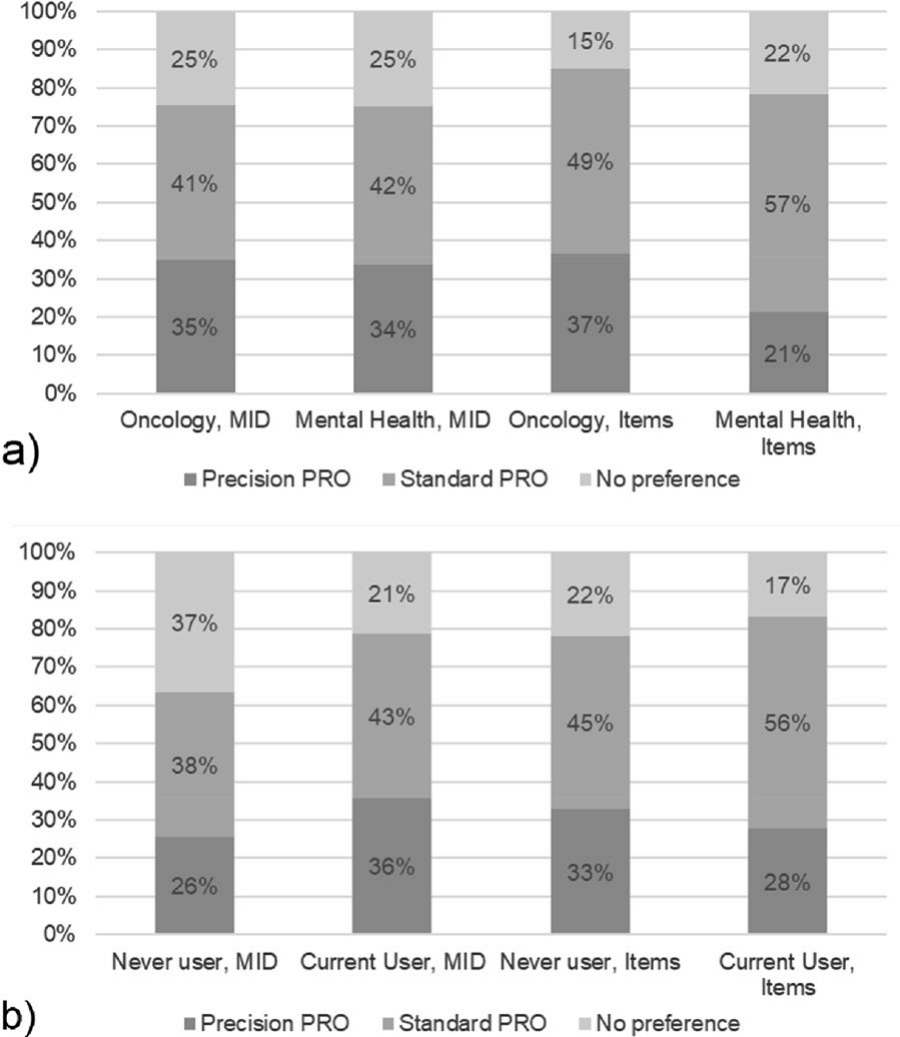
Preferences between individualized (precision) and standard PROs by provider type (**a**) and user type (**b**). The ‘no preference’ category included people who preferred both types, neither type or chose not to answer. Items refers to approach for choosing items. MID = minimally important difference and refers to approach for defining MID

When asked directly about the two different aspects of the Precision PRO approach, between 21 to 37% of each subgroup (oncology provider, mental health provider, never user, current user) expressed a preference for the Precision PRO approach (Fig. 2). Provider type (oncology vs. mental health) was not related to preference for the Precision PRO method of tailoring the MID (p’s > 0.05). Being an oncology provider versus a mental health provider was associated with lower odds of expressing no preference (odds ratio (OR) = 0.445, p = 0.007) or a preference for standardized, nomothetic PROs (OR = 0.478, p = 0.001) compared to the individualized Precision PRO approach of tailoring items. Never users were more likely to express no preference compared to current users (OR = 2.351, p = 0.009) for the Precision PRO method of tailoring the MID, but otherwise user status was unrelated to preferences for Precision PROs vs. standard, nomothetic PROs (p’s > 0.05).

### Qualitative analysis

#### Individualized items

Results from qualitative analyses for the individualized items of the Precision PRO approach are reported in Table 2. Of the 450 respondents, 46 (10.2%) skipped the text question and 20 (4.4%) provided answers that were not codable. Most participants described a preference of PRO version within three main categories: one version is easier or simpler (n = 124), one version is too complicated (n = 42), and the Precision PRO approach to items captures patient variability (n = 69). Some participants cited that they had no preference of PRO version or that they needed more information (n = 34) to decide what was best for their practice. Overall, participants who preferred the standard PRO approach to items (n = 240) said it was simpler and easier. Those who chose the Precision PRO approach to items (n = 126), favored it because it was more individualized and had more patient variability. Other participants found PROs ineffective in general and some MHPs thought patients shouldn’t chose items.

**Table 2:** Qualitative categories for individualized items

Category	Definition	Illustrative quote for those preferring standardized	Illustrative quote for those preferring individualized	Illustrative quote for those with no preference
Easier or simpler (*n* = 53 OP, *n* = 71 MHP)	One version is simpler, easier to complete or shorter (less time consuming)	n = 118 "Simpler and easier to compare"	n = 6 “Simpler for patient to understand”	n = 0
Too complicated (*n* = 20 OP, *n* = 22 MHP)	Converse of "Easier or Simpler" where one of the options seems more complicated or difficult	n = 42 "Version 2 is too complicated and results are difficult to interpret."	n = 0	n = 0
Variability (*n* = 39 OP, *n* = 30 MHP)	Version 2 captures patient variability or what is important to the patient better. Also, Version 1 is not individualized enough	N = 0	n = 69 "More variability"	n = 0
Confusion (*n* = 6 OP, *n* = 3 MHP)	Indicates that person misunderstood description such as assuming Version 1 was short because Version 2 had 30 items. Also, if they say something like more variability in the survey means it is harder to interpret or can't compare Version 2 across patients or across time	n = 4 "Less likely chance of patient fatigue of answering 30 questions and easy, efficient"	n = 4 “Too [m]uch variability in option 2. Hard to compare to prior scores if they choose different 30”	n = 1 “30 questions would be burdensome for some patients and won't complete so might use the shorter one but [question] validity with it”
Want to compare patients (*n* = 16 OP, *n* = 22 MHP)	One version is preferred because it allows comparison of patients	n = 36 "Being able to compare results across all patients"	n = 2 “Using version 2 seems to be favorable for comparison with different patients”	n = 0
Don't want to compare patients (*n* = 2 OP, *n* = 9 MHP)	Do not want to compare patients or find that useful	n = 0	n = 2 “Patient's needs and issues are unique and not comparable to other patients.”	n = 9 "Do not Compare scores between patients"
Need more data (*n* = 1 OP, *n* = 6 MHP)	Need more information about the options to make a decision	n = 0	n = 0	n = 7 "Would need to see the specific forms"
Equal (*n* = 14 OP, *n* = 22 MHP)	No preference	n = 0	n = 0	n = 36 "Both address the issues"
Other option not listed (*n* = 1 OP, *n* = 3 MHP)	Prefer something else besides the two options listed	n = 0	n = 0	n = 4 " We use computer-adaptive technology and the PROMIS validated tool "
Patients are more accurate (*n* = 2 OP, n = 4 MHP)	Patients would be more accurate in reporting symptoms with one version	n = 3 ”I think the results would be more comparable and accurate”	n = 3 "Patient most likely to answer more accurate"	n = 0
Prefer clinical interview or don't see the value (*n* = 4 OP, *n* = 4 MHP)	Do not prefer either because they prefer clinical judgment or interview	n = 1 ”focused care during visit”	n = 0	n = 7 "Takes too much time. Better to just ask patient."
Patients don't want to complete PROs (*n* = 1 OP, *n* = 1 MHP)	Prefers neither because patients don't want to complete PROs in general	n = 0	n = 0	n = 2 "Patients at our clinic do not take these PROs seriously and often get upset having to complete them"
More information (*n* = 21 OP, *n* = 13 MHP)	One version would provide more or better information than the other	n = 3 ”it focuses on most relevant problems, toxicities, response to treatment”	n = 31 "I believe that more useful information would be obtained from version 2 although version 1 would also be helpful."	n = 0
Currently how it’s done (*n* = 2 OP, *n* = 3 MHP)	This is how it has been done before or is the current standard of practice	n = 5 "Because currently how it is done"	n = 0	n = 0
Better Results (*n* = 11 OP, *n* = 5 MHP)	One version would provide better symptom assessment or be easier to interpret	n = 8 "It will be easier to interpret"	n = 8 “If all patients get the same questions, then it is likely that they won't be asked about the information they are interested in giving feedback about.”	n = 0
Patients should not be the one's choosing (*n* = 0 OP, *n* = 4 MHP)	Patients are not able to choose what is important to them or do not know what is most relevant to them	n = 4 "I don't think patients choosing the applicable questions is a good idea, as they may not be able to interpret what applies best to them"	n = 0	n = 0

*OP* oncology provider, *MHP* mental health provider. Standardardized Items was listed as Version 1 and Precision (Individualized) Items was listed as Version 2

#### Individualized MID

Results from qualitative analyses for individualized, Precision PRO MID approach are reported in Table 3. Of the 450 respondents 50 (11.1%) skipped the text questions and 44 (9.8%) provided answers that were not codable. Most participants described a preference of PRO version within four main categories: one version is easier or simpler (n = 82), the Precision PRO captures patient variability (n = 59), one version is perceived as less biased (n = 45), and one version uses patient input (n = 41). Most participants preferred the standard PRO (n = 185) because it was less complicated. However, those who chose the Precision PRO (n = 154), explained that it had more variability and was more individualized for the patient. Some participants did not think patients would know what is meaningful (n = 16). Other participants either did not have a preference between the two PRO versions, or they found PROs ineffective in general.

**Table 3:** Qualitative categories for individualized minimally important difference (MID) comparison (n = 415)

Category	Definition	Illustrative quote for those preferring standardized	Illustrative quote for those preferring individualized	Illustrative quote for those with no preference
Easier or simpler (*n* = 40 OP, *n* = 42 MHP)	One version is simpler, easier to complete, more efficient or shorter (less time consuming). Includes not wanting to interact with the patient	n = 76 "It is simpler"	n = 6 “Easier to in[t]erp[r]et”	n = 0
Too complicated (*n* = 17 OP, *n* = 13 MHP)	Converse of "Easier or Simpler" where one or both of the options seems complicated or difficult	n = 18 ”Version 4 difficult to ascertain use.”	n = 0	n = 12 "Both are confusing and difficult to interpret for both provider and patient"
Better Results (*n* = 14 OP, *n* = 11 MHP)	One version would provide better symptom assessment or be easier to interpret or compare patients	n = 7 ”easier to compare and evaluate for validity of the PRO.”	n = 18 "Better for interpretation"	n = 0
Equal (*n* = 20 OP, *n* = 19 MHP)	No preference or each have merit in different situations	n = 0	n = 0	n = 39 "I feel that both version would provide useful information."
Variability (*n* = 20 OP, *n* = 39 MHP)	Version 4 captures patient variability or what is important to the patient better. Also, Version 3 is not individualized enough or the clinician prefers to have control over meaningful response definition	n = 2 ”Patient decisions differ based on different reasons”	n = 57 "Allows for individualized responses"	n = 0
Confusion (*n* = 2 OP, *n* = 2 MHP)	Indicates that person misunderstood description	n = 3 "I dont think arbitrary "5" difference is meaningful"	n = 0	n = 1 “Likely easier to follow”
Need more data (*n* = 2 OP, *n* = 2 MHP)	Need more information about the options to make a decision	n = 1 ”do not understand #4”	n = 0	n = 3 "Not enough description"
Objective (*n* = 22 OP, *n* = 23 MHP)	One version is perceived as less biased or more standardized	n = 42 "Much more objective"	n = 2 “less patient bias”	n = 1 “too time consuming and subjective”
Patient centered (*n* = 20 OP, *n* = 21 MHP)	One version uses patient input whereas the other does not	n = 1 ”Patient decisions differ based on different reasons”	n = 40 "Patient centered"	n = 0
Patients should not be the one's choosing (*n* = 7 OP, *n* = 9 MHP)	Patients aren't able to choose what's important to them or don't know what is most relevant to them or may be inconsistent. Also included are reasons to not use a patient-defined method like norms are already established	n = 14 "[Patient] won't do a good job deciding this"	n = 1 “di[s]cussing personally helps to dig into whats going on, rather than just getting an answer and hence establish whether its truly cor[r]elated vs its emotional connection”	n = 1 “if patients scores it, there a lot of variation in perception of their symptoms (stoic patients will always undertstate pain for example). Maybe its best to let physician score depending on patient answers”
Prefer clinical interview or don't see the value (*n* = 3 OP, *n* = 2 MHP)	Do not prefer either because they prefer clinical judgment or interview	n = 0	n = 0	n = 5 "I prefer clinical interview"
Foster Collaboration (*n* = 8 OP, *n* = 12 MHP)	Encouraging a discussion would be helpful or one fosters buy in	n = 0	n = 20 "Results will be more accurate and promotes a dialogue with the provider and patient"	n = 0
Patients don't want to complete PROs (*n* = 0 OP, *n* = 1 MHP)	Prefers neither because patients don't want to complete PROs in general	n = 0	n = 0	n = 1 "Patients do not take these PROs seriously and often complain about having to complete them"
Scale is not meaningful (*n* = 9 OP, *n* = 7 MHP)	Don't agree with 5 point change on Version 3 or think the 0–100 scale is terrible	n = 0	n = 2 “0–100 scales are pretty arbitrary. It will be hard for a patient to be consistent from time to time so as to know if a 5 point difference is significant.”	n = 14 "Range of 100 is too broad/vague"
Want to compare patients (*n* = 4 OP, *n* = 5 MHP)	One version is preferred because it allows comparison of patients or promotes uniformity, sometimes for comparison	n = 8 "Able to compare with different patients"	n = 1 “Helps with uniformity amongst patients”	n = 0

*OP* oncology provider, *MHP* mental health provider. Standard MID was listed as Version 3 and Precision (Individualized) MID was listed as Version 4

#### Confusion

Qualitative analysis revealed that some participants found the four versions of PROs to be confusing. The most frequent reasons for confusion were that some participants did not understand the difference of the PRO versions, while others made contradictory remarks to their PRO version selection. Those who stated that they were confused stated they would need more information about the specifics for each version of PRO.

## Discussion

This study examined preferences for individualized PRO measures, such as our Precision PRO method, comparing oncology providers to mental health providers, and comparing never users to current users of PROs. Quantitative results suggested that oncology providers had a more positive general attitude towards standardized measures over individualized measures when compared to mental health providers but oncology providers preferred part of the individualized precision PRO approach, specifically tailoring items, compared to mental health providers. This discrepancy in results for oncology providers could be due to the measures used. The attitude questions were fairly general whereas the preference questions provided specific methods for tailoring PROs within the Precision PRO approach. Some professions like oncology may generally prefer standardized measures but be open to tailoring on specific aspects of PROs, such as which items to administer. Never users may also prefer individualized measures to standardized measures. The Precision PRO approach was preferred by one fifth to one third of the sample, suggesting this approach should be investigated further for patient preference and clinical utility. Qualitative results suggested provider preference was due to either a desire for simplicity or to capture the unique aspects of each patient.

Previous research has shown a preference for individualized measures among mental health providers [8]. Our results, comparing mental health and oncology providers, suggest that how measures are individualized may need to differ by discipline. For oncology, having patients tailor the PRO items may be most important whereas for mental health, having the clinician tailor the items or using standardized items may be most important. However, the overall assessment of individualized measures was neither positive nor negative and, given that standardized measures are what is typically used in clinical care [13], healthcare providers may become more open to individualized measures as they learn more and research progresses.

The limitations of this study warrant comment. This was a convenience sample of mostly physicians. There may have been a responder bias for current PRO users to respond given the high rate of current use in the sample. A subset of respondents also reported confusion about some of the questions and the discrepant results for oncology suggests healthcare providers may have had some difficulty understanding the idea of tailoring PROs.

## Conclusions

Despite the limitations, our results suggest directions for further research on individualized PROs. First, this study only examined provider preference, showing some preference for individualized PROs, but ultimately patient preference is paramount and needs to be examined in future studies. This study showed discipline might moderate provider preference for individualized PROs but did not examine the utility of these measures. Additional research is needed to determine how individualized PROs, such as Precision PROs, compare to standard PROs in predicting outcomes such as mortality and whether individualized PROs can improve clinical care and research.

## Supplementary Information

**Additional file 1.** Supplemental Material.

## Data Availability

De-identified data is available upon reasonable request.
