# Supplementary material for: A comparison of oncologist versus mental health provider attitudes towards standardized and tailored patient-reported outcomes
Source: J Patient Rep Outcomes. 2021 Aug 24;5:76. doi: 10.1186/s41687-021-00352-8 (PMC8384935; doi:10.1186/s41687-021-00352-8)
Supplement: Supplementary file 1 — Additional file 1. Supplemental Material. [file 41687_2021_352_MOESM1_ESM.docx]

Supplemental Material

Survey Text and Questions

Patient-reported outcomes are self-report questionnaires, measures or scales that assess a patient or client’s symptoms or level of function. Patient-reported outcomes are often used in medical care (including behavioral healthcare) to track whether a treatment is working for a particular patient.

Preference Questions

Next, we would like you to review descriptions of different PROs and chose which one you would most like to use in your clinical practice.

PRO version 1:

- 1. Asks patients to rate their symptoms on a 1 (never or rarely) to 5 (always or almost always) scale
  2. Has five items and all patients answer the same five items. Scores from one patient can be compared to scores from another patient.

PRO version 2:

1. Asks patients to rate their symptoms on a 1 (never or rarely) to 5 (always or almost always) scale
2. Patients choose the five items most applicable to them personally from 30 possible items. If items are chosen from the 30 possible items, scores from one patient can be compared to another patient even if each patient uses different items.

Which PRO would you most likely use in your clinical practice?

1. Version 1
2. Version 2
3. Both equally
4. Neither
5. Don’t know

Please tell us briefly the reason for your answer:

PRO version 3:

1. All patients answer the same five items about their symptoms
2. Scores range from 0 to 100. An increase or decrease of five points is considered meaningful for all patients

PRO version 4:

1. All patients answer the same five items about their symptoms
2. Scores range from 0 to 100. Patients define for themselves, sometimes in consultation with their provider, what increase or decrease in symptoms is meaningful for the patient personally.

Which PRO would you most likely use in your clinical practice?

1. Version 3
2. Version 4
3. Both equally
4. Neither
5. Don’t know

Please tell us briefly the reason for your answer:
